# Optimizing Expectations Leads to Enhanced Efficacy of a Mindfulness‐Based Intervention in Individuals With Low‐Baseline Expectations: A Randomized Controlled Trial

**DOI:** 10.1002/jclp.70194

**Published:** 2026-07-21

**Authors:** Stefan Salzmann, Frank Euteneuer, Christiane A. Melzig, Winfried Rief, Christoph Benke

**Affiliations:** ^1^ Clinical Psychology and Psychotherapy Marburg University Marburg Germany; ^2^ Faculty of Human Sciences, Division of Clinical Psychology and Psychotherapy Vinzenz Pallotti University Vallendar Germany; ^3^ Faculty of Human Sciences, Division of Biological Psychology and Neuroscience Vinzenz Pallotti University Vallendar Germany; ^4^ Clinical Psychology, Experimental Psychopathology and Psychotherapy Marburg University Marburg Germany

**Keywords:** mindfulness, moderator, placebo effect, stress, tailored treatment, treatment expectation

## Abstract

**Introduction:**

Treatment expectations for medical and psychological treatments and positive patient‐provider interactions are critical mechanisms driving the placebo effect and may augment an intervention's effects. However, it remains unclear whether optimizing treatment expectations is beneficial for all individuals. This pilot randomized controlled trial (three‐arm parallel design) examined whether i) optimizing treatment expectations would augment the efficacy of a mindfulness intervention and ii) whether participants' treatment expectations at baseline would moderate these effects.

**Methods:**

Sixty‐six healthy participants were randomized (1:1:1) to one of 3 experimental groups: i) body scan and an instruction aiming to optimize treatment expectations (EXPECT), ii) body scan and a neutral instruction (NEUTRAL), or iii) listening to an audiobook and neutral instruction (CONTROL). Primary outcomes were changes in positive and negative affect; secondary outcomes included treatment expectations, perceived stress, and mindfulness. Baseline expectations were assessed as a moderator using ANCOVA.

**Results:**

ANCOVA indicated a group effect on positive affect (*p* = 0.018, d = 0.65), with individuals in the EXPECT group showing a stronger reduction than both other groups. Post‐hoc comparisons showed significant differences between EXPECT and NEUTRAL (*p* = 0.015, d = −0.77) and between EXPECT and CONTROL (*p* = 0.012, d = −0.80). Analyzes did not indicate main effects on other outcomes. Participants with low baseline expectations showed a stronger increase in reported treatment expectations and mindfulness, as well as a more pronounced decrease in perceived stress from baseline‐ to post‐assessment in the EXPECT group compared to both other groups. In contrast, individuals with high baseline expectations indicated less stress reduction when undergoing the body scan and receiving the instruction with positive expectation induction (EXPECT) compared to the group with a body scan and neutral instruction (NEUTRAL).

**Conclusion:**

Inducing positive treatment expectations may be beneficial for individuals with low baseline expectations, likely due to an enhanced placebo response. However, it may be counterproductive for individuals with high baseline outcome expectations and could reflect expectation violation effects.

## 1 Introduction

The field of experimental placebo research has advanced significantly, with robust evidence demonstrating that mechanisms such as an individual's expectations play a pivotal role in driving placebo effects and, consequently, treatment outcomes (Petrie and Rief 2019; Schedlowski et al. 2015). Despite these advances, the translation of placebo‐related insights into clinical practice remains limited, as structured approaches to systematically assess and optimize patient expectations are rarely implemented in routine care, clinical guidelines on how to actively harness placebo mechanisms remain scarce, and there is limited guidance on how such mechanisms can be tailored to individual patient characteristics (Colloca et al. 2023). Additionally, negative expectations can contribute to nocebo effects, which refer to adverse symptoms or outcomes arising from such expectations, and may prevent optimal treatment outcomes or even cause side effects and harm (Colloca and Finniss 2012). Therefore, managing patients' expectations may provide opportunities for optimizing therapeutic results without altering the medical or psychological core interventions. However, it remains unclear how to best manage expectations in clinical settings.

Importantly, a growing body of research has specifically investigated the role of expectation and placebo effects in mindfulness interventions using balanced placebo or sham‐controlled designs. These studies aim to disentangle mindfulness‐specific effects from nonspecific factors such as treatment expectations. For example, experimental studies have shown that expectation manipulations can substantially influence cognitive outcomes such as interference control (stroop task) or verbal fluency of brief meditation interventions, sometimes even exceeding the effects of the meditation itself (Prätzlich et al. 2016). Similarly, recent randomized controlled trials using sham mindfulness conditions suggest that improvements following brief mindfulness interventions may be substantially influenced by nonspecific factors such as expectations, rather than being solely attributable to mindfulness‐specific processes (Bos et al. 2024; Davies et al. 2023). In these studies, sham mindfulness conditions typically involve structurally similar exercises (e.g., listening tasks, breathing instructions without mindfulness‐specific guidance, or attention control tasks) that mimic key features of the intervention while omitting its putative active components. These findings align with broader placebo research demonstrating that treatment effects and expectation effects may interact in non‐additive ways, highlighting the importance of considering both components when evaluating and optimizing intervention efficacy (Bingel 2020; Coleshill et al. 2018).

Positive baseline treatment expectations significantly influence psychobiological outcomes ‐ such as symptom perception (e.g., pain), stress responses, and treatment‐related neural activity (e.g., brain responses in pain‐processing networks) ‐ following an intervention through both physiological and psychological mechanisms (Bingel 2020; Kube et al. 2018; Petrie and Rief 2019). Therefore, optimizing these expectations presents a promising approach to enhancing treatment outcomes. First results indicate that optimizing patients’ expectations before medical treatments leads to improved outcomes (e.g., Akroyd et al. 2020; Rief et al. 2017). However, a key challenge in this area is the variability in individual responses, where an intervention that works for one person may be ineffective or even counterproductive for another. Previous research has shown the importance of interindividual differences such as personality traits, inflammatory status, or depressive symptoms in placebo and nocebo responses (Horn et al. 2022; Kern et al. 2020; Salzmann et al. 2021). These findings highlight the importance of considering individual differences when examining how placebo mechanisms can be clinically harnessed to improve outcomes.

Although baseline expectations are crucial predictors of outcomes, no studies have specifically explored how these expectations may moderate the effects of interventions designed to leverage placebo mechanisms. This gap is significant in understanding whether optimizing expectations benefits all individuals equally or if more tailored approaches are necessary. This study seeks to examine whether enhancing outcome expectations prior to a brief psychological intervention can increase its efficacy and whether individuals' pre‐existing expectations influence the efficacy of this optimization.

Mindfulness‐Based Interventions (MBIs), such as Mindfulness‐Based Stress Reduction (MBSR), have demonstrated promising effects across various populations and outcomes (Benavides‐Gil et al. 2024; Burton et al. 2017; Yakobi et al. 2021). However, the high time demands required by multi‐week programs often discourages participation (Gan et al. 2022). As a result, there is growing interest in utilizing single components of these comprehensive programs. This approach, known as single‐session interventions (SSIs), provides a practical and resource‐efficient strategy, especially in settings where time and resources are limited (Schleider and Beidas 2022; Schleider and Weisz 2017).

The body scan is considered an introductory and accessible component of MBSR, often one of the first exercises practiced in‐depth (Colgan et al. 2016; Kabat‐Zinn 2010). A meta‐analysis found that a single body scan session produced only small effects on mindfulness, but not on other outcomes compared to a passive control condition (Gan et al. 2022). Given that single‐session mindfulness interventions demonstrated measurable effects but still have significant room for improvement, and the mechanisms underlying expectation optimization are not exclusive to mindfulness interventions, the body scan serves as an ideal model to examine how expectations influence intervention efficacy.

This pilot study primarily aimed to determine whether a brief positive expectation induction could enhance the efficacy of MBIs. Based on previous studies on the effects of body scans, we hypothesized that the group with expectation optimization would show a stronger reduction in positive and negative affect, a stronger reduction in perceived stress and a stronger increase in mindfulness compared to both other groups.

Recent evidence also highlights how individual characteristics can shape the outcomes of MBIs, with studies showing that participants' baseline mindfulness levels or psychological traits can moderate treatment effects (Buric et al. 2022; Sieder et al. 2024). However, while various baseline traits have been investigated, the specific role of baseline treatment expectations in moderating the effects of mindfulness interventions remains unexplored. Therefore, as a secondary objective of this study, we investigated if individuals with lower baseline expectations experience greater benefits in affect, perceived stress, and mindfulness compared to those with higher expectations. Our rationale was that individuals with initially lower expectations might benefit from an increase in expectations, whereas in individuals with already high expectations, further enhancing expectations may create a mismatch between anticipated and actual experiences, potentially limiting benefits or even leading to unfavorable or counterproductive effects, as suggested by expectation violation models (Rief et al. 2022). This tailored approach aligns with the movement toward personalized medicine, which aims to optimize therapeutic outcomes by adapting interventions to individual characteristics (Enck et al. 2013).

Especially improving the initial experience of an SSI may help increase long‐term adherence. Given that lifestyle‐related factors significantly contribute to morbidity and mortality (Martinez et al. 2020), interventions that promote stress reduction and overall well‐being and especially the knowledge who may benefit from an expectation optimization to boost intervention effects may be of substantial value. A deeper understanding of these dynamics could inform more effective, personalized interventions, enhancing benefits while minimizing risks such as nocebo effects.

## 2 Materials and Methods

### 2.1 Participants

This pilot study included 66 healthy participants, primarily university students. Participants were recruited through online advertisements and were eligible if they were at least 18 years old and were fluent in German. We used the following exclusion criteria to reduce the sample heterogeneity: Individuals with severe cardiovascular diseases (e.g., post‐myocardial infarction, treatable hypo‐ or hypertension) or neurological conditions (e.g., epilepsy, stroke, multiple sclerosis) were not permitted to participate in the study. Individuals who were on continuous medication (excluding contraceptives, dietary supplements, and l‐thyroxine for hypothyroidism) were also not allowed to participate. In the case of pregnancy, the study was deemed unsuitable. No explicit exclusion criteria regarding mental health conditions were applied. However, symptoms of depression and anxiety were assessed at baseline using the Patient Health Questionnaire (PHQ)‐4 to characterize baseline psychological distress and account for potential confounding influences. All participants provided verbal and written informed consent prior to participation. As an incentive for participation, subjects received 1.5 h of credit towards research participation. Alternatively, participants had the option to enter a raffle for a chance to win one of five vouchers, each valued at €20.

We conducted an a priori power analysis using G*Power 3.1 for an F test of a repeated‐measures ANOVA with a within‐between interaction. Assuming an effect size of f = 0.20, α = 0.05, power (1–β) = 0.80, 3 groups, and 2 measurement time points, the required total sample size was *N* = 66. The power analysis was based on the primary interaction of interest (group × time), while the final analyzes used ANCOVA models adjusting for baseline values, which provide a statistically equivalent and often more powerful approach in randomized pre‐post designs. The recruitment was stopped after randomizing 66 participants.

#### 2.1.1 Design, Randomization, and Procedure

This randomized controlled trial (three‐arm parallel design) was conducted as a single‐session laboratory study to investigate whether treatment expectations modulate the efficacy of a mindfulness‐based intervention. The local Ethics Committee approved the study protocol. This study was registered at www.clinicaltrials.gov (NCT05115240). The experimental study took place between September 2, 2021, and November 3, 2021. Participants were recruited via online advertisements and mailing lists and, if eligible, asked in a short interview about the predefined in‐ and exclusion criteria. Those participants who met the requirements were invited to the University Department. The examination room contained a desk with a computer, where participants completed the questionnaire portion of the study. Additionally, the room had another desk where the experimenter remained during data collection. The study consisted of a single laboratory visit lasting approximately 60–90 min, including baseline assessment, intervention, and post‐intervention measures.

In adherence to constant environmental conditions, the lighting remained on during each session. All participants, as well as the experimenter, wore medical masks during the study due to hygiene measures implemented in response to the COVID‐19 pandemic. After reading the study information and signing the informed consent form, participants completed the baseline assessments, including measures of affect, stress, mindfulness, and treatment expectations. All questionnaires were completed via SoSciSurvey (Leiner 2019). The randomization sequence was generated using Randomizer (https://www.randomizer.org/). Envelopes containing the allocation sequence were prepared in advance by staff members not involved in the experiment. While the experimenter was not blinded to the treatment condition upon opening the envelopes, participants remained blinded to their assigned condition and the existence of other study arms until the experiment concluded. After the baseline assessment, participants were randomized (1:1:1) to one of the three experimental manipulations:

1. **EXPECT Group:** Participants read an instruction designed to optimize positive treatment expectations before undergoing a body scan exercise (the full instructions can be found in the supplemental material).
2. **NEUTRAL Group:** Participants read a neutral instruction before receiving the same body scan as the EXPECT group, serving as an active control.
3. **CONTROL Group:** Participants read a neutral instruction before listening to an audiobook, serving as an additional control.

#### 2.1.2 Intervention and Expectation Manipulation

The EXPECT and NEUTRAL groups both completed a standardized 22:55‐min body scan exercise with background music provided by the German health insurance provider Techniker Krankenkasse (Frobeen 2021). The EXPECT group's instruction (read before undergoing the body scan) emphasized the scientifically proven benefits of the body scan—such as relaxation, stress reduction, mood enhancement, and immune support—aiming to elevate (positive) treatment expectations. Jon Kabat‐Zinn, who integrated the body scan into his stress‐reduction program, was cited as an expert to support these claims. In contrast, the NEUTRAL group received a straightforward, factual description of the body scan process without attempting to enhance expectations, providing only a factual description of the procedure, detailing the sequence of attention from the feet upwards through the body.

The CONTROL group, by comparison, listened to a neutral instruction before hearing an excerpt from *The Hobbit* by J.R.R. Tolkien. This control condition was used to isolate content effects, matching the duration and setting of the mindfulness interventions without any mindfulness‐related content.

To ensure consistency, participants in all groups were seated on a chair and were instructed to close their eyes if they wished, observing thoughts and sensations without judgment. Identical environmental conditions—seating, length, lighting, and word count—were maintained across groups, with only the EXPECT group's expectation‐optimizing content differing within the mindfulness conditions. This setup allowed for a focused analysis of how expectation modification influences mindfulness outcomes, as well as a comparison between mindfulness and non‐mindfulness interventions.

The experimenter, positioned 2.5 meters from the participants, maintained a friendly yet neutral demeanor throughout the session. Following the baseline assessment, participants were randomly assigned to conditions before reading the respective instructions. After reading the instructions, they rated their treatment expectations a second time, completed either the body scan or audiobook intervention, and immediately repeated all subsequent assessments. The body scan intervention began with grounding instructions and proceeded through a comprehensive full‐body mindfulness sequence.

### 2.2 Outcome Measures

The primary outcomes of the study were changes in positive and negative affect, while treatment expectations, perceived stress, and mindfulness were secondary outcomes.

Participants' affect was assessed using the Positive and Negative Affect Schedule (PANAS) (Watson et al. 1988), a widely used and well‐validated measure of affect with good internal consistency and construct validity (Crawford and Henry 2004). The PANAS is a self‐report questionnaire that consists of two 10‐item scales to measure both positive and negative affect. Each item is rated on a 5‐point scale of 1 (not at all) to 5 (very much). Scores for positive and negative affect were computed as the mean of the respective items, resulting in a scale range from 1 to 5. Higher scores indicate higher levels of positive affect, whereas higher scores on the negative affect scale indicate higher levels of negative affect. The PANAS has been shown to be sensitive to short‐term fluctuations in affective states, making it suitable for assessing pre–post changes in intervention studies (Thompson 2007). Change scores were calculated as post minus pre values, resulting in a theoretical range from −4 to +4. Positive values indicate an increase, and negative values indicate a reduction in positive affect following the intervention.

Participants' treatment expectations were assessed using a single‐item measure on a 6‐point scale ranging from 0 (“not at all”) to 5 (“very much”). The item was designed to capture participants' expectations regarding the efficacy of the mindfulness exercise and read: “What do you expect, how much can a short exercise help you improve your mood?”. Single‐item measures are frequently used to assess state‐like expectations in experimental and placebo research, where expectations are conceptualized as context‐dependent and momentary constructs (e.g., Salzmann et al. 2022). Given the brief and situational nature of the construct, a single‐item measure was considered appropriate to minimize participant burden and capture immediate expectations. However, it should be noted that single‐item measures may be limited in terms of reliability and do not allow for internal consistency estimation. Change scores were calculated as post minus pre values, resulting in a theoretical range from −5 to +5. Positive values indicate an increase, and negative values indicate a reduction in treatment expectations following the intervention.

Perceived stress was assessed using a visual analog scale (VAS), where participants rated their current stress level from 0 (“not stressed at all”) to 100 (“extremely stressed”). VAS measures are widely used to assess momentary subjective states and are considered sensitive to short‐term fluctuations in experimental contexts. A similar approach has been used in prior expectation‐based intervention studies (e.g., Salzmann et al. 2022), supporting its suitability for capturing transient changes in perceived stress. As with other single‐item measures, this approach prioritizes low response burden but does not permit the assessment of internal consistency. Change scores were calculated as post minus pre values, resulting in a theoretical range from −100 to +100.

The Toronto Mindfulness Scale (TMS) (Lau et al. 2006) was used to assess mindfulness as a state rather than a trait, which allows it to measure changes in mindfulness following an intervention. The TMS was specifically developed to capture fluctuations in mindfulness resulting from meditation practice and has demonstrated good internal consistency and construct validity (Lau et al. 2006). We assessed state mindfulness rather than trait mindfulness since a state measure would theoretically be better able to capture the effects of a single, short intervention compared to trait measures. The scale consists of 13 positively worded items that participants rate on a five‐point Likert scale. The five‐point response scale ranges from “not at all” (1) to “very much” (5). The TMS has a two‐factor structure, assessing the dimensions of curiosity and decentering. These factors are calculated by summing the responses to specific items: curiosity is measured by items 3, 5, 6, 10, 12, and 13, while decentering is measured by the remaining items. For the curiosity subscale, scores range from 6 to 30, and for the decentering subscale from 7 to 35. Change scores were calculated as post minus pre values resulting in theoretical ranges from −24 to +24 for curiosity and −28 to +28 for decentering.

The Patient Health Questionnaire‐4 (PHQ‐4) is a brief screening tool assessing symptoms of anxiety and depression, with good reliability and construct validity across diverse populations (Kroenke et al. 2009). Comprised of four items, it includes a 2‐item scale each for anxiety (GAD‐2) and depression (PHQ‐2). Each item is scored on a 4‐point scale (0–3), with a total score ranging from 0 to 12. Scores are categorized to indicate levels of psychological distress: 0–2 for no distress, 3–5 for mild, 6–8 for moderate, and 9–12 for severe distress.

### 2.3 Statistical Analysis

Analyzes were performed using ANCOVA models to assess the main effects of different interventions and the interaction between baseline expectations and group assignment on the outcomes. Specifically, the models tested whether changes in the outcomes from pre‐ to post (change scores were calculated by subtracting the baseline (pre) scores from the post scores as the dependent variable) differed between the three intervention groups (main effects). All outcome measures were assessed at baseline and immediately after the intervention (body scan or audiobook). To assess the manipulation effect, treatment expectations were additionally measured after the instruction (and before the intervention). Baseline scores of each outcome measure were included as covariates to control for individual differences. In a second step, interaction terms between group and baseline expectations were used to explore whether individuals' baseline expectations moderate the intervention effects. Significant interactions were followed by post‐hoc simple slope analyzes to better understand the effects at different levels of baseline expectations [low (‐1 SD), average (mean), or high (+ 1 SD)].

In addition to its descriptive use, baseline psychological distress (PHQ‐4 total score) was included in exploratory analyzes as a potential moderator of intervention effects by testing interaction terms between group and PHQ‐4 scores in the ANCOVA models.

A significance level of *p* < 0.05 was used to determine statistical significance. Effect sizes are reported as Cohen's *d*, derived from adjusted group differences estimated in the ANCOVA models. Values of 0.20, 0.50, and 0.80 indicate small, medium, and large effects, respectively. All statistical analyzes were conducted using SPSS version 29. Since this study was a pilot trial, we did not correct for multiple testing.

## 3 Results

A total of 66 participants (77% female, mean age = 35.6 years, SD = 17) were randomized (Figure 1) into three groups: EXPECT (*n* = 22), NEUTRAL (*n* = 22), and CONTROL (*n* = 22). One person in the EXPECT group was excluded from the analysis due to the premature discontinuation of the intervention, resulting in *N* = 65 participants.

**Figure 1:**
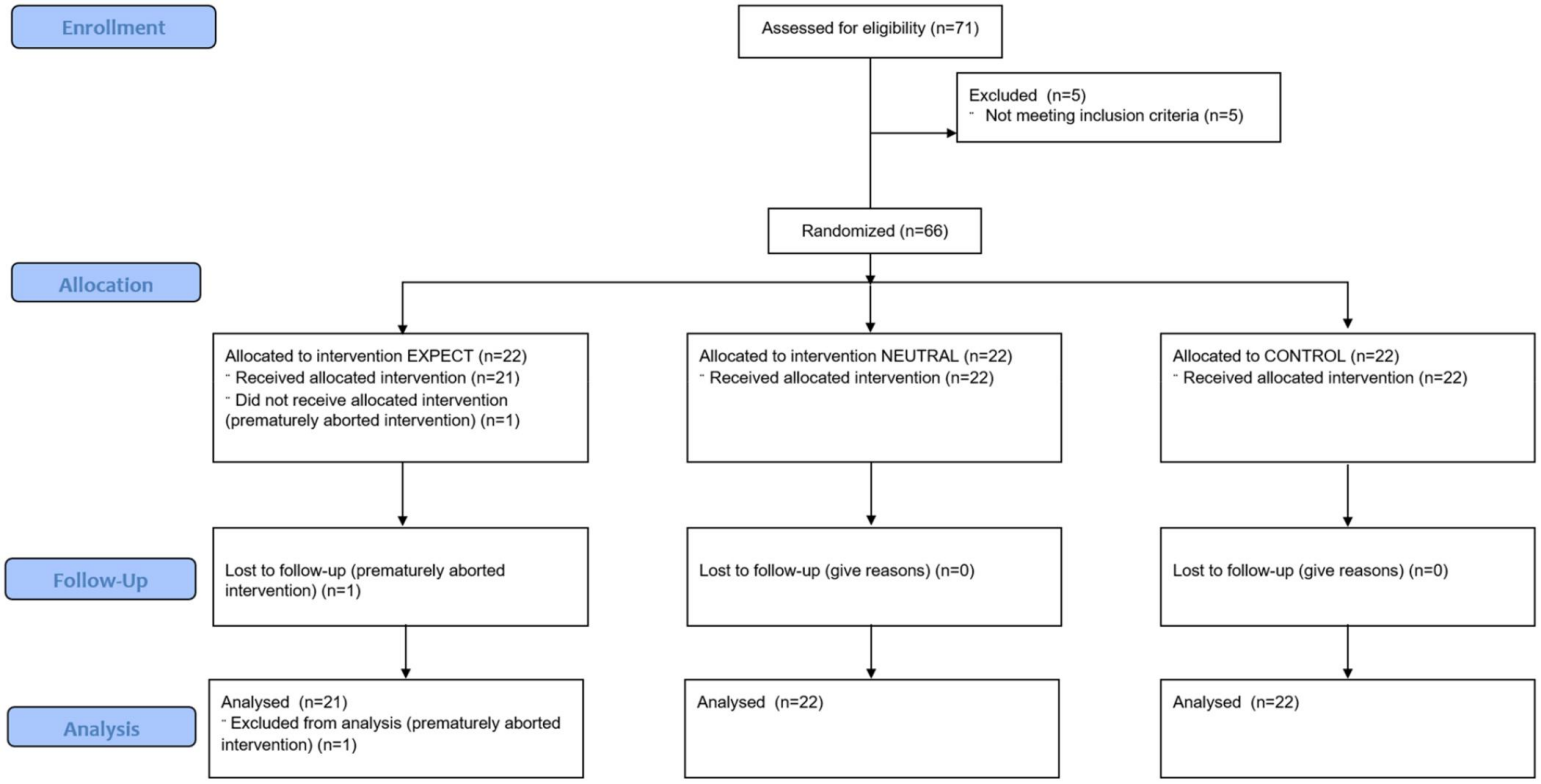
CONSORT flowchart.

The groups were comparable regarding baseline levels of perceived stress, mindfulness, and treatment expectations (Table 1).

**Table 1:** Baseline sample characteristics.

	EXPECT (*n* = 21)	NEUTRAL (*n* = 22)	CONTROL (*n* = 22)
Age, *mean years (SD)*	36.1 (16.7)	32.8 (17.6)	38.0 (17.2)
Gender, female *n (%)*	14 (66.6)	20 (90.9)	17 (77.3)
Baseline outcome expectations regarding the benefits of MBI, *mean (SD)*	2.9 (0.89)	3.41 (0.96)	2.95 (1.05)
Baseline perceived stress, *mean (SD)*	15.0 (18.62)	10.5 (15.53)	13.23 (16.39)
Experience with MBI or audiobook (yes), *n (%)*	17 (81)	19 (86.4)	8 (36.4)
Baseline positive affect (PANAS), *mean (SD)*	3.11 (0.46)	3.23 (0.59)	2.90 (0.58)
Baseline negative affect (PANAS), *mean (SD)*	1.17 (0.16)	1.26 (0.25)	1.24 (0.25)
Baseline mindfulness curiosity (TMS), *mean (SD)*	14.1 (4.81)	19.68 (4.41)	13.77 (6.38)
Baseline mindfulness decentering (TMS), *mean (SD)*	19.9 (3.73)	20.82 (3.86)	17.55 (5.55)
Baseline depressive symptoms (PHQ‐2), *mean (SD)*	2.76 (0.83)	2.18 (1.01)	2.32 (1.25)
Baseline anxiety symptoms (GAD‐2), *mean (SD)*	2.52 (0.81)	2.45 (1.18)	2.36 (1.43)
Baseline psychological distress (PHQ‐4), *mean (SD)*	5.29 (1.31)	4.64 (1.84)	4.68 (2.38)

Abbreviations: GAD, generalized anxiety disorder; MBI, mindfulness‐based intervention; PANAS, positive and negative affect schedule; PHQ, patient health questionnaire; TMS, Toronto mindfulness scale.

### 3.1 Main Effects of the Intervention

The analyzes indicated no statistically significant group difference for NA (F(2, 61) = 0.181, *p* = 0.835, d = 0.13), stress (F(2, 61) = 0.207; *p* = 0.814, d = 0.14), expectations (F(2, 61) = 2.614; *p* = 0.081, d = 0.51), or mindfulness (curiosity: F(2, 61) = 2.042; *p* = 0.13, d = 0.446; decentering: F(2, 61) = 2.390; *p* = 0.10, d = 0.48, Table 2). However, the analyzes indicated significant group differences regarding the change of PA from pre‐ to post‐assessment indicated by a main effect of intervention group (F(2, 61) = 4.317, *p* = 0.018, d = 0.65): Individuals in the EXPECT group showed a stronger reduction in positive affect compared to both other groups (EXPECT vs. NEUTRAL: *p* = 0.015, d = −0.77; EXPECT vs. CONTROL: *p* = 0.012, d = −0.80). The two other groups did not show a significantly different change in PA (NEUTRAL vs. CONTROL: *p* = 0.917, d = −0.03) Figure 2.

**Table 2:** Pre‐ and post‐intervention outcomes by group.

Outcome	Group	Pre mean (SD)	Post mean (SD)	Δ	F	*p*	*d*
Positive affect (PANAS)	EXPECT	3.11 (0.46)	2.35 (0.58)	−0.76	4.317	0.018	0.65
	NEUTRAL	3.23 (0.59)	2.91 (0.84)	−0.32			
	CONTROL	2.90 (0.58)	2.67 (0.78)	−0.23			
Negative affect (PANAS)	EXPECT	1.17 (0.16)	1.04 (0.09)	−0.13	0.181	0.835	0.13
	NEUTRAL	1.26 (0.25)	1.08 (0.13)	−0.18			
	CONTROL	1.24 (0.25)	1.08 (0.14)	−0.16			
Treatment expectations	EXPECT	2.90 (0.89)	3.43 (0.75)	+0.53	2.614	0.081	0.51
	NEUTRAL	3.41 (0.96)	3.36 (1.14)	−0.05			
	CONTROL	2.95 (1.05)	2.91 (0.75)	−0.04			
Perceived stress	EXPECT	26.52 (25.00)	15.00 (18.62)	−11.52	0.207	0.814	0.14
	NEUTRAL	20.82 (19.77)	10.50 (15.53)	−10.32			
	CONTROL	22.18 (22.13)	13.23 (16.39)	−8.95			
Mindfulness (Curiosity, TMS)	EXPECT	14.10 (4.81)	18.57 (5.16)	+4.48	2.042	0.130	0.45
	NEUTRAL	19.68 (4.41)	21.91 (4.73)	+2.23			
	CONTROL	13.77 (6.38)	16.09 (5.70)	+2.32			
Mindfulness (Decentering, TMS)	EXPECT	19.90 (3.73)	23.86 (2.59)	+3.96	2.390	0.100	0.48
	NEUTRAL	20.82 (3.86)	24.05 (4.20)	+3.23			
	CONTROL	17.55 (5.55)	20.14 (5.88)	+2.59			

*Note:* PANAS (Positive and Negative Affect Schedule), TMS (Toronto Mindfulness Scale). Pre‐ and post‐values are unadjusted means (SD). Test statistics are based on ANCOVAs controlling for baseline levels of the outcome.

**Figure 2:**
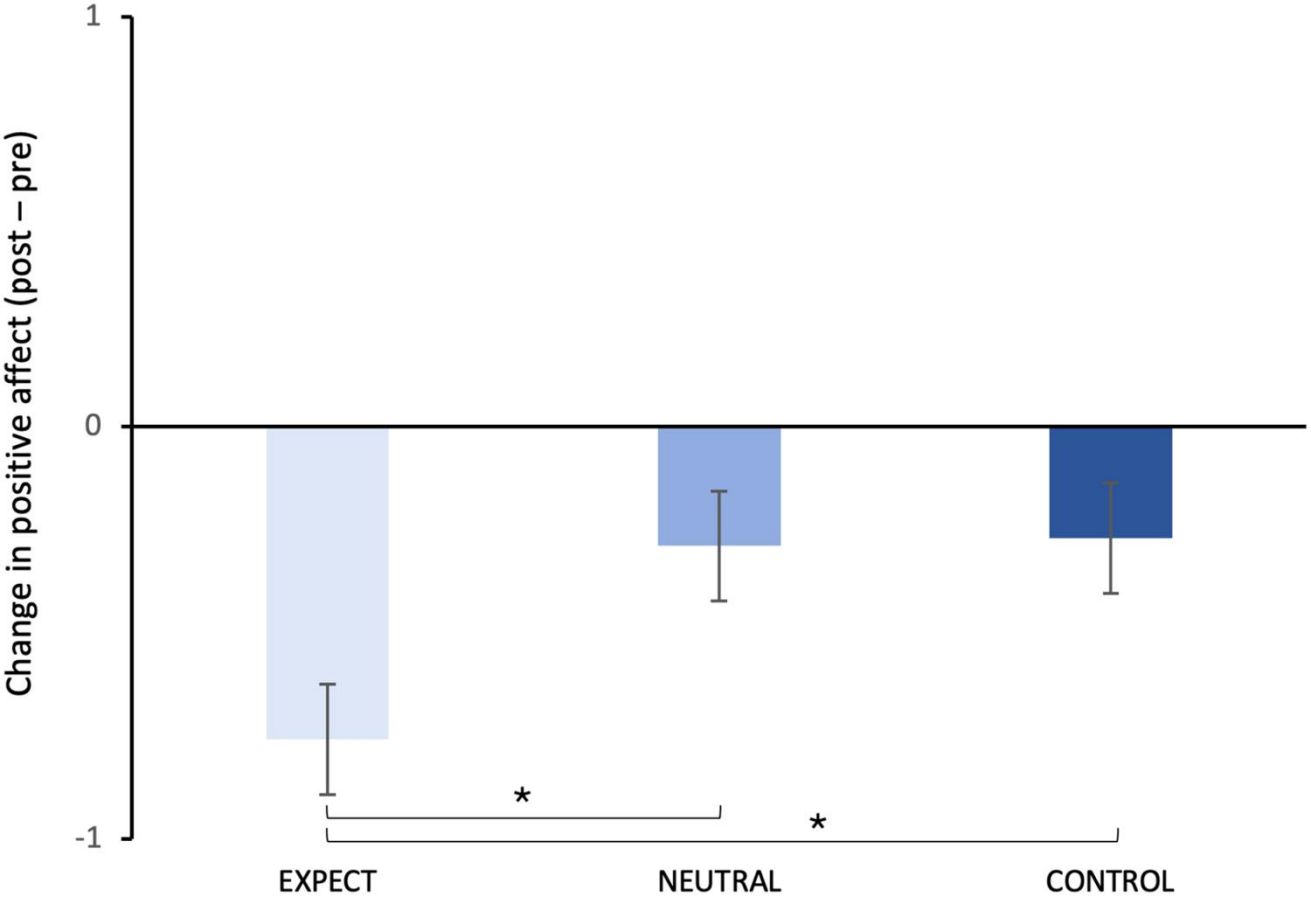
Change in positive affect. Positive affect was assessed using the PANAS (mean score across 10 items; range: 1–5). Change scores were calculated as post minus pre values, resulting in a theoretical range from −4 to +4. Positive values indicate an increase, and negative values indicate a reduction in positive affect following the intervention. Error bars represent ± standard error of the mean (SEM). * indicates statistically significant pairwise differences between groups (EXPECT vs. NEUTRAL; EXPECT vs. CONTROL) at *p* < 0.05.

### 3.2 Change in Treatment Expectations Depending on the Level of Baseline Expectations

Baseline treatment expectation moderated the intervention effects on participants' expectations as indicated by a significant interaction between group and baseline expectations (F(2, 59) = 5.532, *p* = 0.006, d = 0.74; Figure 3): Participants with low baseline expectations demonstrated an increase from baseline to post‐assessment for all groups. However, the EXPECT group exhibited a significantly greater increase in treatment expectations from baseline to post‐intervention compared to both other groups (EXPECT vs. NEUTRAL: *p* = 0.002, d = 0.99, EXPECT vs. CONTROL: *p* = 0.032, d = 0.67, NEUTRAL vs. CONTROL: *p* = 0.179, d = 0.41). In individuals with average baseline expectations, the EXPECT intervention resulted in a significantly higher increase in expectations from baseline to post‐assessment than the CONTROL group (*p* = 0.027, d = 0.70) and a trend for higher values compared to the NEUTRAL group (*p* = 0.095, d = 0.52; NEUTRAL vs. CONTROL: *p* = 0.595, d = 0.16). Individuals with high baseline expectations showed an expectation reduction in all groups with significantly larger decreases in the CONTROL group compared to the NEUTRAL group (*p* = 0.018, d = 0.73). All other post‐hoc group comparisons were non‐significant (EXPECT vs. NEUTRAL: *p* = 0.321, d = −0.31, EXPECT vs. CONTROL: *p* = 0.263, d = 0.35).

**Figure 3:**
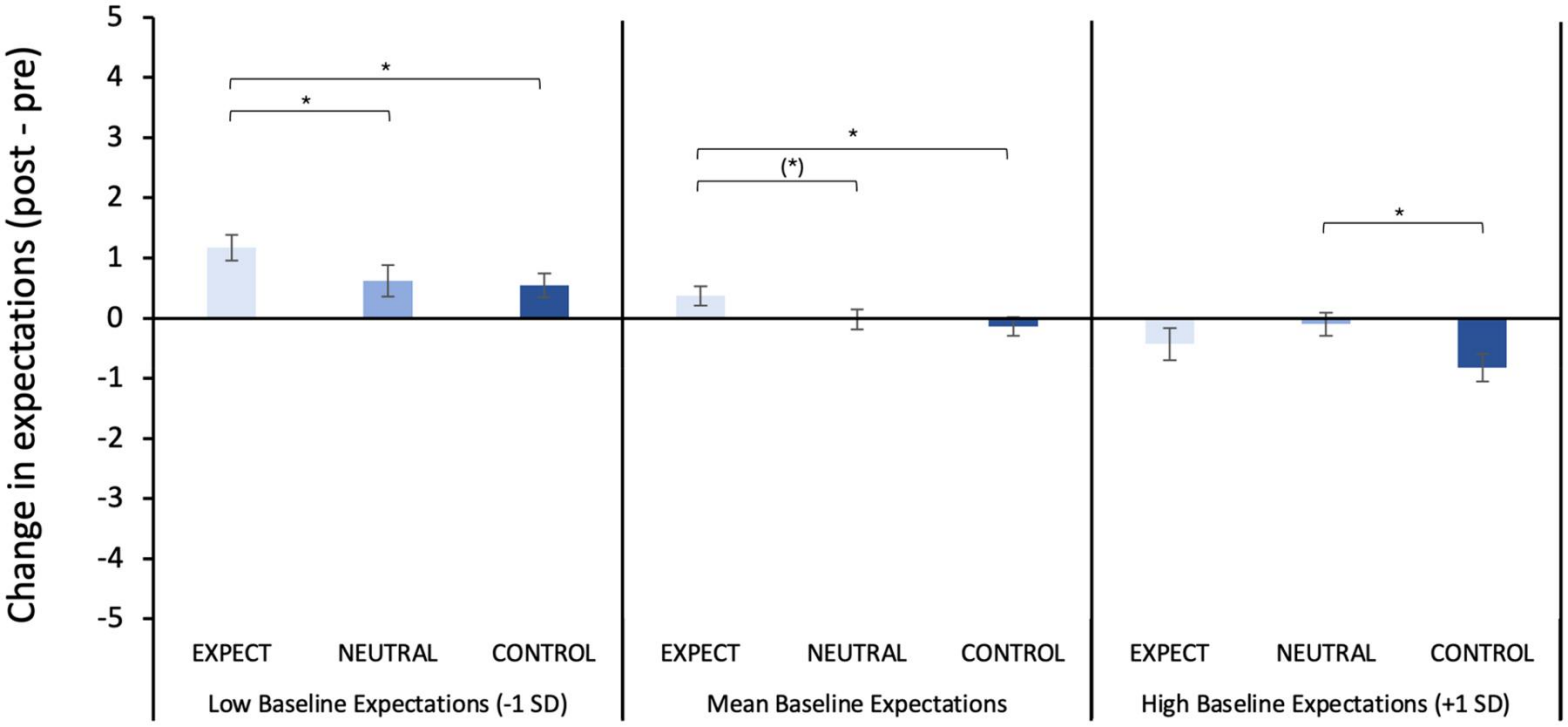
Change in treatment expectations by baseline expectation levels. Treatment expectations were assessed using a single‐item measure ranging from 0 to 5. Change scores were calculated as post minus pre values, resulting in a theoretical range from −5 to +5. Positive values indicate an increase, and negative values indicate a reduction in treatment expectations following the intervention. Error bars represent ± standard error of the mean (SEM). * indicates statistically significant pairwise differences between groups (EXPECT vs. NEUTRAL; EXPECT vs. CONTROL) at *p* < 0.05, while (*) indicates *p* < 0.10.

### 3.3 Change in Perceived Stress Depending on the Level of Baseline Expectations

Baseline expectations moderated the intervention effects on participants' perceived stress levels (F(2, 58) = 5.875, *p* = 0.005, d = 0.90; Figure 4): In participants with low baseline expectations, the EXPECT group demonstrated a significantly greater reduction in perceived stress from baseline to post‐intervention compared to NEUTRAL (*p* = 0.043, d = −0.64) and a trend towards a greater reduction compared to CONTROL (*p* = 0.054, d = −0.60, NEUTRAL vs. CONTROL: *p* = 0.697, d = 0.12). In contrast, participants with high baseline expectations showed a significantly smaller reduction in perceived stress in the EXPECT group compared to the NEUTRAL group (*p* = 0.013, d = 0.79) and a trend towards a smaller reduction compared to the CONTROL group (*p* = 0.52, d = 0.61, NEUTRAL vs. CONTROL: *p* = 0.612, d = −0.15). For individuals with average baseline treatment expectations, there were no significant group differences (EXPECT vs. NEUTRAL: *p* = 0.799, d = 0.08, EXPECT vs. CONTROL: *p* = 0.822, d = 0.07, NEUTRAL vs. CONTROL: *p* = 0.972, d = −0.01).

**Figure 4:**
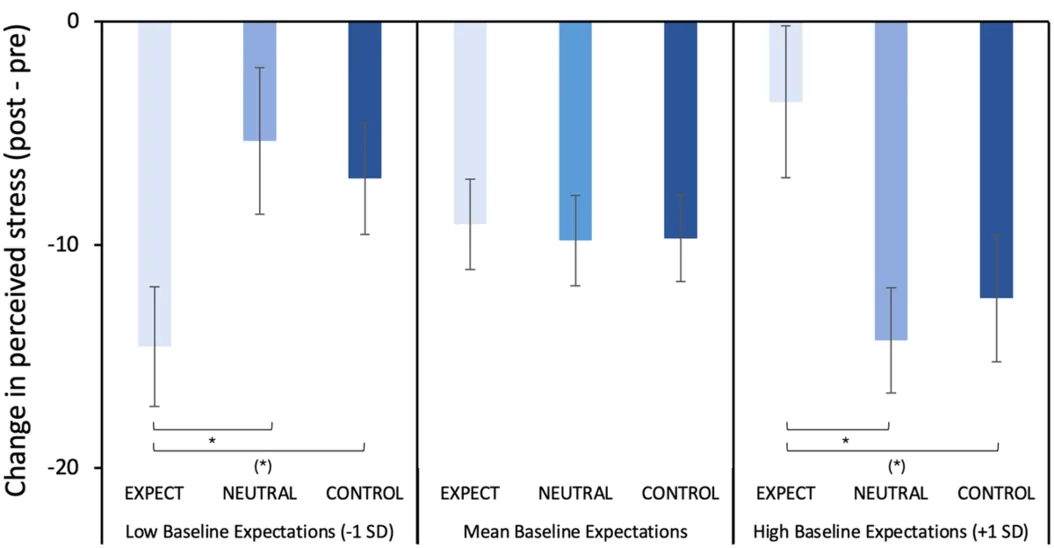
Change in perceived stress by baseline expectation levels. Perceived stress was assessed using a visual analogue scale (VAS; range: 0–100). Change scores were calculated as post minus pre values, resulting in a theoretical range from −100 to +100. For improved interpretability, the y‐axis is displayed on a restricted range. Negative values indicate a reduction in perceived stress following the intervention. Error bars represent ± standard error of the mean (SEM). * indicates statistically significant pairwise differences between groups (EXPECT vs. NEUTRAL; EXPECT vs. CONTROL) at *p* < 0.05, while (*) indicates *p* < 0.10.

### 3.4 Change in Negative Affect Depending on Baseline Expectations

Analyzes indicated a significant interaction between group and baseline expectations for NA (F(2, 58) = 3.577, *p* = 0.034, d = 0.70; Figure 5). For participants with low baseline expectations, those in the EXPECT group tended to have stronger decreases in NA compared to NEUTRAL (*p* = 0.054, d = −0.61) and CONTROL (*p* = 0.071, d = −0.57; NEUTRAL vs. CONTROL: *p* = 0.726, d = 0.11). Post‐hoc tests indicated no group differences for individuals with average (EXPECT vs. NEUTRAL: *p* = 0.680, d = −0.13, EXPECT vs. CONTROL: *p* = 0.834, d = −0.07, NEUTRAL vs. CONTROL: *p* = 0.832, d = 0.07) or high baseline expectations (EXPECT vs. NEUTRAL: *p* = 0.137, d = 0.47, EXPECT vs. CONTROL: *p* = 0.182, d = 0.42, NEUTRAL vs. CONTROL: *p* = 0.942, d = −0.02).

**Figure 5:**
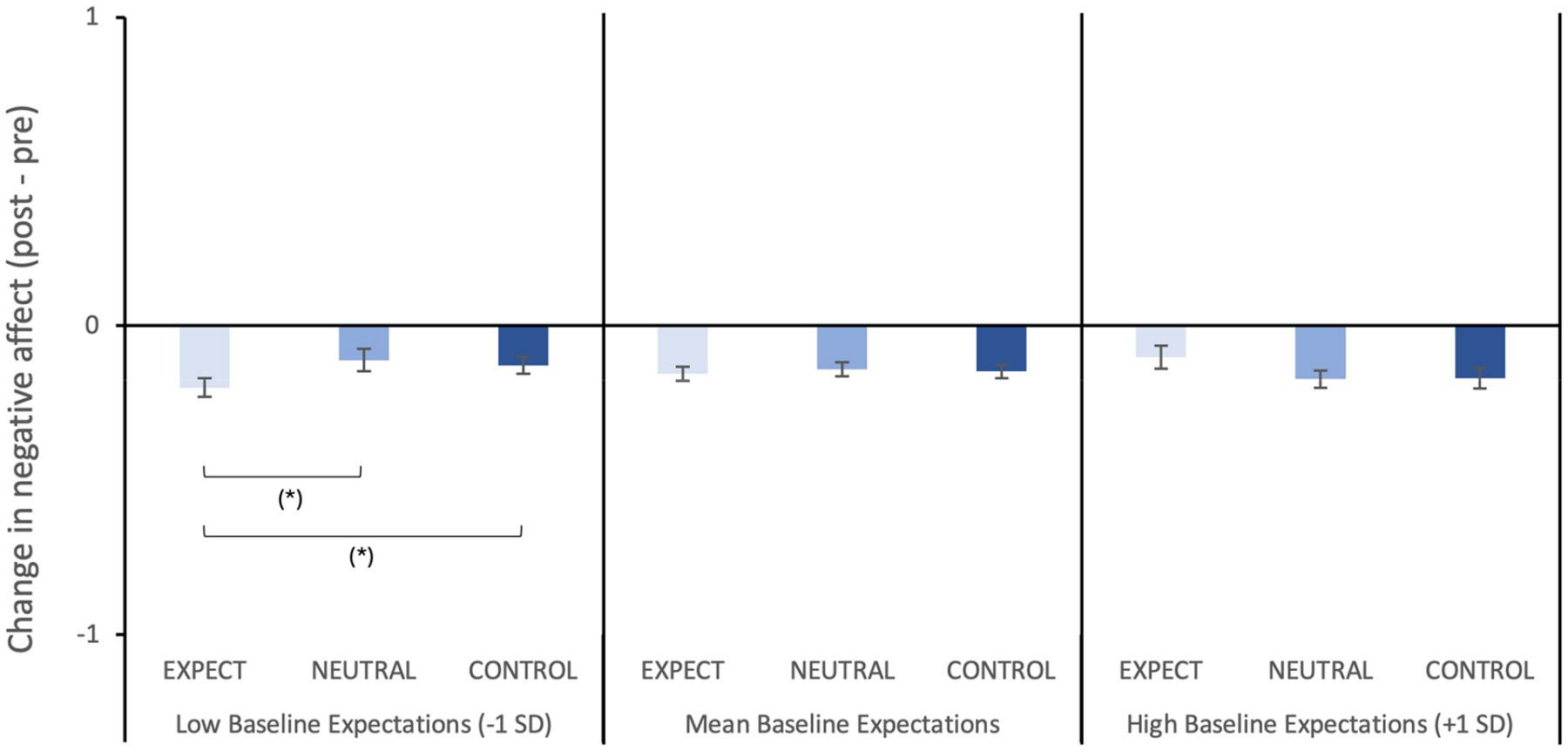
Change in negative affect by baseline expectation levels. Negative affect was assessed using the PANAS (mean score across 10 items; range: 1–5). Change scores were calculated as post minus pre values, resulting in a theoretical range from −4 to +4. For improved interpretability, the y‐axis is displayed on a restricted range. Negative values indicate a reduction in negative affect following the intervention. Error bars represent ± standard error of the mean (SEM). * indicates statistically significant pairwise differences between groups (EXPECT vs. NEUTRAL; EXPECT vs. CONTROL) at *p* < 0.05, while (*) indicates *p* < 0.10.

### 3.5 Change in Mindfulness Depending on Baseline Expectations

The intervention effects on the mindfulness curiosity subscale were significantly moderated by participants' baseline expectations (F(2, 58) = 10.853; *p* < 0.001, d = 1.22; Figure 6). Participants with low baseline expectations benefit from the EXPECT intervention in terms of significantly stronger increases in curiosity from pre‐to post‐assessment compared to both other groups (EXPECT vs. NEUTRAL: *p* = 0.002, d = 1.00, EXPECT vs. CONTROL: *p* < 0.001, d = 1.13, NEUTRAL vs. CONTROL: *p* = 0.803, d = −0.08). For individuals with high baseline expectations, those in the NEUTRAL condition indicated significant stronger increases in curiosity from pre‐to post‐assessment compared to EXPECT (*p* = 0.004, d = −0.93), and a trend towards stronger increases compared to CONTROL (*p* = 0.051, d = 0.60), but no significant difference between EXPECT and CONTROL (*p* = 0.262, d = −0.35). There were no significant differences for individuals with average baseline treatment expectations (EXPECT vs. NEUTRAL: *p* = 0.713, d = 0.11, EXPECT vs. CONTROL: *p* = 0.131, d = 0.47, NEUTRAL vs. CONTROL: *p* = 0.315, d = 0.30). The intervention effects were not moderated by participants' baseline expectations for mindfulness subscale decentering (F(2, 58) = 2.327; *p* = 0.107, d = 0.57).

**Figure 6:**
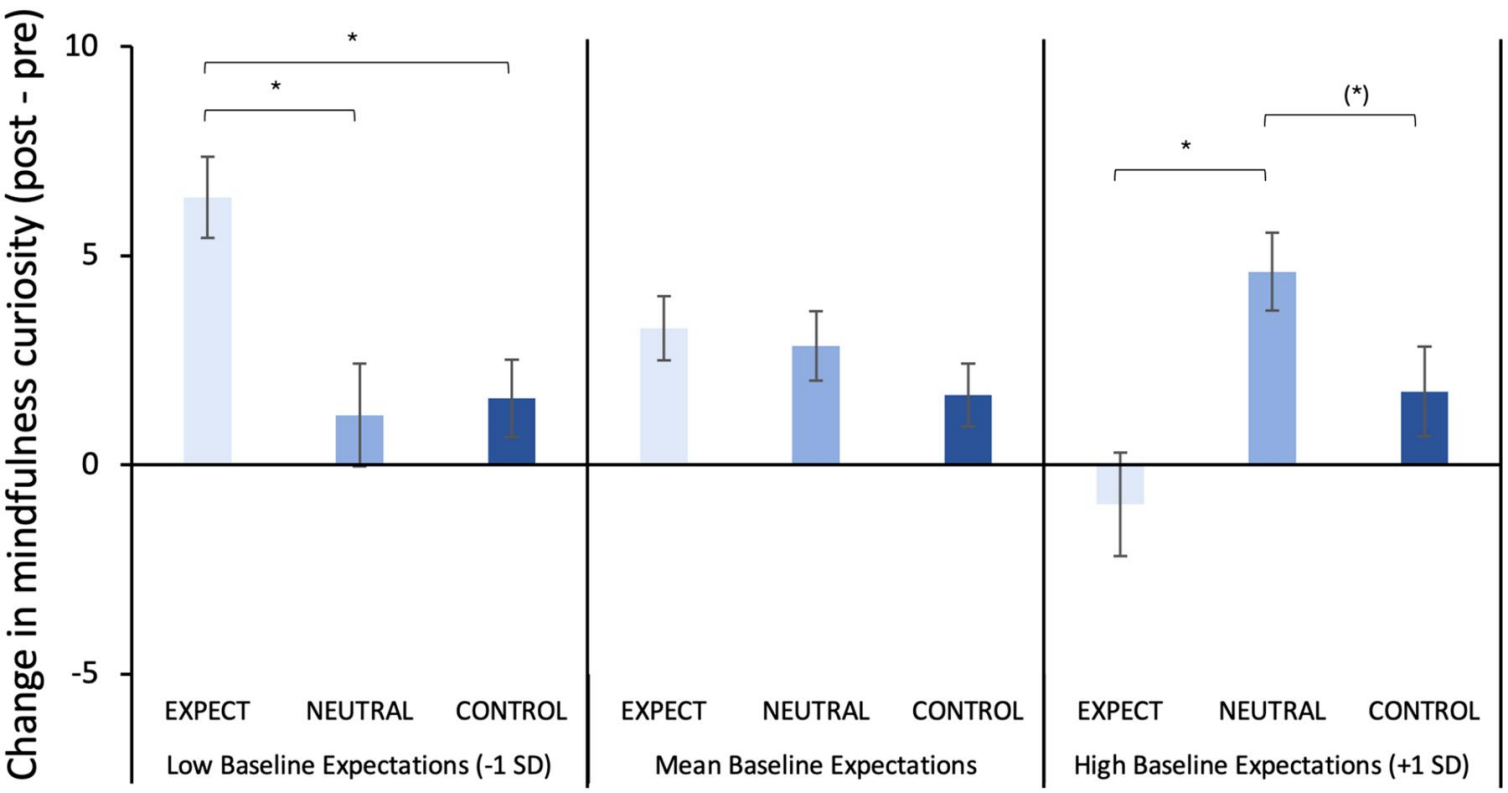
Change in mindfulness (curiosity) by baseline expectation levels. Mindfulness (curiosity) was assessed using the Toronto Mindfulness Scale (TMS; curiosity subscale, sum score across 6 items). The TMS consists of 13 items rated on a five‐point Likert scale ranging from “not at all” (1) to “very much” (5) and comprises two subscales (curiosity and decentering). The curiosity subscale ranges from 6 to 30. Change scores were calculated as post minus pre values, resulting in a theoretical range from −24 to +24. For improved interpretability, the y‐axis is displayed on a restricted range. Positive values indicate an increase in mindfulness (curiosity) following the intervention. Error bars represent ± standard error of the mean (SEM). * indicates statistically significant pairwise differences between groups (EXPECT vs. NEUTRAL; EXPECT vs. CONTROL) at *p* < 0.05, while (*) indicates *p* < 0.10.

### 3.6 Change in Positive Affect Depending on Baseline Expectations

The intervention effects were not moderated by participants' baseline expectations for PA (F(2, 58) = 0.910, *p* = 0.408, d = 0.35).

### 3.7 Additional Analyzes

Participants' treatment expectations after the intervention correlated significantly with the change in perceived stress from baseline to post‐intervention (r = −0.248, *p* = 0.046), but not with change in PA (r = −0.046, *p* = 0.715), NA (r = −0.124, *p* = 0.324), or mindfulness (curiosity: r = 0.16, *p* = 0.203 decentering: r = −0.029, *p* = 0.819). The change in treatment expectations from baseline to post‐manipulation correlated with the change in PA (r = −0.288, *p* = 0.02) and mindfulness (curiosity: r = 0.276, *p* = 0.026), but not with the change in NA (r = −0.038, *p* = 0.761) or mindfulness (decentering: r = 0.032, *p* = 0.797).

To examine whether baseline psychological distress moderated intervention effects, interaction terms between group and PHQ‐4 scores were included in ANCOVA models controlling for baseline levels of the respective outcome variables. Baseline distress did not significantly moderate intervention effects on PA, NA, treatment expectations, perceived stress, or mindfulness (all *p* ≥ 0.089). Although effect sizes for some outcomes (e.g., treatment expectations, decentering) were in the small‐to‐moderate range (| d | = 0.17–0.59), these effects did not reach statistical significance.

## 4 Discussion

This study aimed to explore whether optimizing treatment expectations could enhance the efficacy of a mindfulness‐based intervention (MBI), particularly investigating the moderating role of baseline expectations. Our findings suggest that inducing positive expectations may enhance intervention outcomes, particularly in individuals with low baseline expectations. However, we also observed that the same strategy may reduce the intervention's efficacy in individuals with high baseline expectations, potentially reflecting expectation violation effects (Rief et al. 2022).

Since we did not observe main effects of group for most outcomes, the present findings do not provide evidence that the body scan was superior to the active control condition in which participants listened to an audiobook. However, the absence of group differences should be interpreted with caution, as this pilot study may have been underpowered to detect small‐to‐moderate effects. Moreover, while the use of an active control condition allowed us to account for several structural and contextual non‐specific factors (e.g., time, attention, and task engagement), the absence of a sham mindfulness condition precludes firm conclusions regarding the specific efficacy of the intervention, as non‐specific factors may still contribute substantially to observed outcomes (Davies et al. 2023). Several factors may help explain the absence of main effects across most outcomes. First, the brevity of the intervention (single‐session body scan) may have been insufficient to produce robust changes beyond those induced by nonspecific factors. Second, although treatment expectations were experimentally manipulated and assessed in the present study, nonspecific influences may not be fully captured by this operationalization. Previous studies using balanced placebo and sham‐controlled designs suggest that outcomes of brief mindfulness interventions can be substantially influenced by a broader set of nonspecific factors, including but not limited to expectations, such as demand characteristics or contextual cues (Bos et al. 2024; Davies et al. 2023; Prätzlich et al. 2016). Third, the NEUTRAL condition may not represent a purely inert comparison, as even neutral instructions may induce expectations or engagement. Finally, the sensitivity of the employed measures may have been insufficient to detect subtle short‐term changes following a brief intervention.

Our results seem to be in contrast with research indicating that MBIs have shown to be efficacious across different outcomes in different samples when being applied over several weeks (Benavides‐Gil et al. 2024; Burton et al. 2017; Yakobi et al. 2021). However, our findings are in line with research showing that more time‐efficient approaches such as single‐session body scans have only been shown to produce small effects on mindfulness, but no effects on other outcomes compared to passive control groups in a meta‐analysis (Gan et al. 2022).

An unexpected finding of the present study was the reduction in positive affect across all conditions, which was most pronounced in the EXPECT group. At first glance, this appears counterintuitive, as both mindfulness interventions and positive expectation inductions are typically associated with improvements in mood. However, this pattern may be partly explained by the characteristics of the measurement instrument used. The PANAS primarily captures high‐arousal positive affect (e.g., excitement, enthusiasm) rather than low‐arousal states such as calmness or relaxation (Watson et al. 1988). It is therefore plausible that the body scan—as well as the audiobook condition—reduced high‐arousal states while simultaneously promoting low‐arousal positive states that were not adequately captured by the measure. This interpretation is consistent with prior research suggesting that mindfulness practices may decrease affective intensity rather than uniformly increasing positive affect, particularly in mindfulness‐naïve individuals (e.g., Palmer et al. 2023).

In addition, the more pronounced reduction in positive affect in the EXPECT group may reflect an expectation–experience mismatch. Participants who received expectation‐enhancing instructions may have anticipated a more salient or immediate improvement in mood, and the absence of such a strong effect may have resulted in a relative deflation of reported affect. This interpretation aligns with theoretical models of expectation violation, which posit that discrepancies between anticipated and experienced outcomes can influence subjective evaluations of interventions (Rief et al. 2022). However, as expectation–experience discrepancies were not directly assessed in the present study, this explanation remains speculative.

From a broader perspective, these findings may also be interpreted within frameworks of placebo–treatment additivity. Expectation effects and intervention‐specific effects do not necessarily combine in a purely additive manner and may instead interact in subadditive or even counteractive ways (Coleshill et al. 2018). In this context, it is possible that strong positive expectations altered the subjective evaluation of the intervention rather than enhancing its affective impact, particularly in individuals whose expectations were not met. Future studies using designs that explicitly manipulate and disentangle expectation and intervention components (e.g., balanced placebo designs) are needed to clarify these mechanisms.

### 4.1 Optimizing Expectations: Benefits and Limitations

For participants with low baseline expectations, the positive expectation induction (EXPECT group) resulted in a substantial increase in treatment expectations, concomitant with a more pronounced reduction in perceived stress and an increase in mindfulness compared to those without expectation optimization. The negative correlation between participants' treatment expectations and stress reduction indicated a direct association between expectations and perceived stress, but not with other outcomes. These findings align with a growing body of placebo literature, which highlights that positive expectations can amplify the effects of both medical and psychological interventions by clinically harnessing placebo mechanisms to boost patients' therapeutic responses after an intervention (Akroyd et al. 2020; Rief et al. 2017). Our study extends this understanding by demonstrating that even brief manipulations of expectations can have meaningful effects on intervention outcomes, especially in individuals with low pre‐existing treatment expectations.

In contrast to individuals with low baseline treatment expectations, participants with high baseline expectations experienced smaller reductions in perceived stress and mindfulness when exposed to the positive expectation induction compared to those who did not receive the EXPECT instruction. This suggests that elevating expectations beyond a certain threshold may create a mismatch between anticipated and actual experiences, potentially leading to an expectation violation effect, whereby such discrepancies may induce a mismatch‐related negative appraisal and reduce perceived intervention efficacy (Rief et al. 2022). This finding is in line with results from a study in patients undergoing psychosomatic rehabilitation, where a positive discrepancy between expectations and experiences related to these outcome expectations as well as a negative discrepancy (meaning that the experiences are more negative compared to the previous expectations) between expectations and experiences related to concerns indicated associations with higher patient satisfaction (Glattacker et al. 2022). In our own study, negatively violated treatment expectations (i.e., when experiences were worse than expected) after surgery were associated with higher pain levels (Zerth et al. 2025). Elevated positive expectations may set unrealistically high standards, and when these are not met, expectation violation processes may influence outcome evaluations and have been associated with worse clinical outcomes (Rief et al. 2022).

Another explanation for this finding may be that individuals with high baseline expectations might simply feel irritated when they are presented with information extolling the benefits of an intervention they already perceive positively. This additional promotion may be perceived as redundant or even condescending, potentially triggering a counterproductive reaction due to psychological reactance, which may apply when people feel that their freedom of behavior is threatened or eliminated due to information received (Rosenberg and Siegel 2018). Such findings underscore the complexity of expectation management in therapeutic contexts and suggest that an indiscriminate elevation of pre‐existing expectations may not always be beneficial.

These findings advocate for a tailored approach to managing patient expectations, aligning with personalized medicine principles (Enck et al. 2013). Specifically, boosting expectations may enhance therapeutic outcomes for individuals with low baseline expectations, while individuals with high baseline expectations might benefit from a neutral or moderate framing of potential benefits. Integrating baseline expectation assessments into clinical practices could thus help optimize intervention efficacy by reducing the risk of expectation violation effects where mismatches between expectations and actual experiences may reduce the perceived efficacy of an intervention or amplify adverse outcomes (Colloca et al. 2023). For instance, the effects of a potent pain‐killer drug could be enhanced when positive expectations were induced, or rendered ineffective due to negative expectations (Bingel et al. 2011) showing the variance regarding a drug's efficacy depending on an individual's psychological state influenced by receiving information prior to a treatment. Our study adds to the literature by showing that inducing positive expectations may be important in people with low treatment expectations; however, it may also be harmful or reduce an intervention's general efficacy in individuals having already high treatment expectations. In individuals with average pre‐existing expectations it may not be helpful but may also not cause harm.

The results have practical implications, probably not only for clinicians administering mindfulness or stress‐reduction therapies. As the study demonstrated, using expectation management based on initial assessments could improve intervention outcomes by fostering realistic and achievable goals. Future clinical protocols could incorporate expectation management techniques as part of intake assessments to align intervention strategies with patients' psychological profiles. Such personalized approaches could enhance engagement, reduce adverse effects, and foster long‐term adherence to mindfulness practices, and may also influence treatment initiation and continuation over time. Further, clinicians should also pay attention to other placebo mechanisms such as a good patient‐provider interaction or previous experiences (Schedlowski et al. 2015).

### 4.2 Limitations and Future Research

Although this study provides valuable insights, several limitations should be noted. The short‐term nature of the intervention limits conclusions on long‐term effects, and future research should investigate whether expectation‐induced benefits or challenges persist over time. Additionally, broadening the study to include diverse psychological and therapeutic interventions would enhance generalizability. Investigating the underlying mechanisms that drive varying responses to expectation induction, such as personality traits or neurobiological factors, could further refine strategies for optimizing therapeutic outcomes. Future research may also benefit from considering trait mindfulness as a potential moderator of intervention effects.

Baseline psychological distress (PHQ‐4) was explored as a potential moderator of intervention effects. Contrary to expectations, distress did not significantly moderate outcomes in the present study. While prior research suggests that depressive and anxiety symptoms may influence placebo and nocebo responsiveness (e.g., Horn et al. 2022; Salzmann et al. 2022), our findings did not provide evidence for such effects in this sample. However, these results should be interpreted with caution given the limited sample in this pilot study, which may have reduced statistical power to detect small‐to‐moderate interaction effects. Future studies with larger and more diverse samples are needed to more conclusively examine the role of psychological distress in moderating expectation‐based intervention effects.

Furthermore, the sample consisted primarily of university students, which may limit the generalizability of the findings to broader clinical or community populations. The relatively small sample size per group may have limited statistical power to detect small‐to‐moderate between‐group and interaction effects, increasing the risk of Type II errors, and the findings should therefore be interpreted with caution. Additionally, the audiobook used in the control condition may not have been entirely neutral, as individual preferences or familiarity with the content could have influenced affective responses. Another important limitation is that, in this study, we only focused on increasing positive treatment expectations regarding the benefits of the body scan. However, other studies show that in some populations (reducing) side‐effect expectations may be even more important than (increasing) positive expectations. Until we have a better understanding of the relevance of different expectations, it would be best, if clinicians consider both positive and negative expectations when suggesting interventions to their patients.

## 5 Conclusion

In conclusion, our study highlights the potential for optimizing treatment expectations to enhance the efficacy of single‐session mindfulness‐based interventions, particularly for individuals with low baseline expectations. However, the results also emphasize the need for a tailored approach, as individuals with high baseline expectations may experience diminished benefits or even adverse outcomes when exposed to (a too) positive expectation induction. These findings underscore the importance of personalized interventions that consider individual psychological profiles to maximize therapeutic outcomes and minimize the risk of expectation violation effects resulting from mismatches between expectations and actual experience. Future research should continue to explore ways to optimize expectation management in psychological therapies, contributing to more precise and effective clinical interventions.

## Conflicts of Interest

The authors declare that the research was conducted in the absence of any commercial or financial relationships that could be construed as a potential conflicts of interest.

## Clinical Trial Registration

The local Ethics Committee of the University of Marburg, Germany approved the study protocol. This study was registered at www.clinicaltrials.gov (NCT05115240).

## Supporting information


Supporting File

## Data Availability

The data that support the findings of this study are available on request from the corresponding author. The data are not publicly available due to privacy or ethical restrictions.
