# Supplementary material for: Optimizing Expectations Leads to Enhanced Efficacy of a Mindfulness‐Based Intervention in Individuals With Low‐Baseline Expectations: A Randomized Controlled Trial
Source: J Clin Psychol. 2026 Jul 21;82(11):1809–21. doi: 10.1002/jclp.70194 (PMC13630349; doi:10.1002/jclp.70194)
Supplement: Supplementary file 1 [file JCLP-82-1809-s001.docx]

**Supplemental Material**

**Instruction EXPECT (English tranlsation)**

Health is of enormous importance in our society, and mindfulness exercises are becoming increasingly valued due to their positive effects on health. One of the most well-known and effective mindfulness exercises is the body scan.

The body scan has already helped many people. Here are just a few examples of its positive effects: it helps reduce stress (even lowering stress hormones) and strengthens the immune system; it increases compassion, which can improve social relationships; it aids in managing pain more effectively and improves sleep quality. For these reasons, the renowned medical professor Jon Kabat-Zinn incorporated the body scan into his stress reduction program (Mindfulness-Based Stress Reduction).

Because of these widely documented positive effects, you will be guided through a body scan in the next 20 minutes.

Many studies have particularly highlighted the positive influence of the body scan on well-being and mood. Positive emotions are enhanced, while negative emotions and stress are experienced less intensely. Awareness of bodily signals is improved, which not only strengthens the perception of positive emotions but also enables the early detection of stress, allowing timely countermeasures.

The body scan can be done while sitting or lying down. In this exercise, the focus is less on your outer posture and more on your inner attitude. Remain attentive and approach all sensations, emotions, and thoughts that you experience with kindness and without judgment. In this short exercise, you will be performing the body scan in a seated position. Please do not be concerned if you are instructed to lie down in the upcoming audio sequence. Simply follow all instructions as if they were intended for a seated position rather than lying down.

**Instruction NEUTRAL (English tranlsation)**

In the next 20 minutes, you will participate in a body journey, known as the body scan. This means you will be guided through different regions of your body, following an audio sequence that will lead you through the body scan.

The body scan is a mindfulness exercise rooted in the Buddhist Vipassana tradition, originally part of the meditation practice of U Ba Khin. Jon Kabat-Zinn integrated the body scan into his eight-week program, known as the Mindfulness-Based Stress Reduction (MBSR) program. In this MBSR program, the body scan is a foundational exercise that prepares participants for other meditation techniques. The body scan is also commonly used in yoga.

The body scan is performed in a comfortable position within a quiet, undisturbed environment. Attention is gradually directed to various parts of the body, starting with a focus on breathing, then moving from the feet, up the legs, through the torso, and finally to the arms and head.

The body scan can be practiced while sitting or lying down. In this exercise, the emphasis is less on your outer posture and more on your inner attitude. Stay alert and approach all sensations, feelings, and thoughts you experience with friendliness and without judgment. In this brief exercise, you will be performing the body scan in a seated position. Therefore, please do not be concerned if you are instructed to lie down in the upcoming audio sequence. Simply follow all instructions as if they were intended for a seated position rather than lying down.

**Instruction CONTROL (English tranlsation)**

This short exercise in this study consists of listening to an audio sequence here in this room for about 20 minutes. This audio sequence is an excerpt from an audiobook. The audiobook is based on the fantasy novel *The Hobbit* by J. R. R. Tolkien, written between 1930 and 1936.

The story begins in the Shire, in the west of the continent of Middle-earth, where the hobbits live. Hobbits are fictional, human-like beings, also known as halflings, as they are about half the size of humans. In the excerpt you are about to hear, the well-known wizard Gandalf the Grey unexpectedly appears in front of the hobbit Bilbo Baggins' home. Gandalf has not been seen in the area for a long time, so Bilbo does not recognize him. The wizard is looking for someone for an adventure, but Bilbo Baggins is not interested. He tells Gandalf that he is looking in the wrong place for an adventurer. Bilbo says goodbye, closes the door, and returns inside his home. Gandalf then quietly marks Bilbo’s door with a symbol and leaves. The next day, Bilbo Baggins is unexpectedly visited by 13 dwarves who invite themselves to eat and drink.

You will listen to the audio sequence while seated due to the attached electrodes. You are welcome to close your eyes during this time. Stay attentive and approach any sensations, thoughts, or feelings that may arise with friendliness and without judgment. Observe them impartially.
